# Unlocking the power of light: Innovative solutions for severe acne—A report of two cases

**DOI:** 10.1111/jocd.16485

**Published:** 2024-07-24

**Authors:** Marc Falguera‐Mayoral, Franco Gemigniani

**Affiliations:** ^1^ Department of Dermatology Vall d'Hebron University Hospital Barcelona Spain; ^2^ Klein Dermatological Clinic Santiago Chile

To the Editor

Acne is an inflammatory condition of the pilosebaceous follicle, affecting millions globally. Despite various topical and oral treatments, some patients exhibit resistance, leading to persistent lesions, scarring, and a decline in quality of life. Certain treatments are contraindicated for specific patients, and some individuals choose to forgo these treatments. Light therapy (LT) has emerged as a viable option in this context.^1^ We present two clinical cases delineating the efficacy of intense pulsed light (IPL) and non‐ablative fractional laser (NFAL) Erbium:Glass 1550 nm (Nordlys System by Candela Medical, USA) in the management of severe acne.

## CASE 1

**FIGURE 1:**
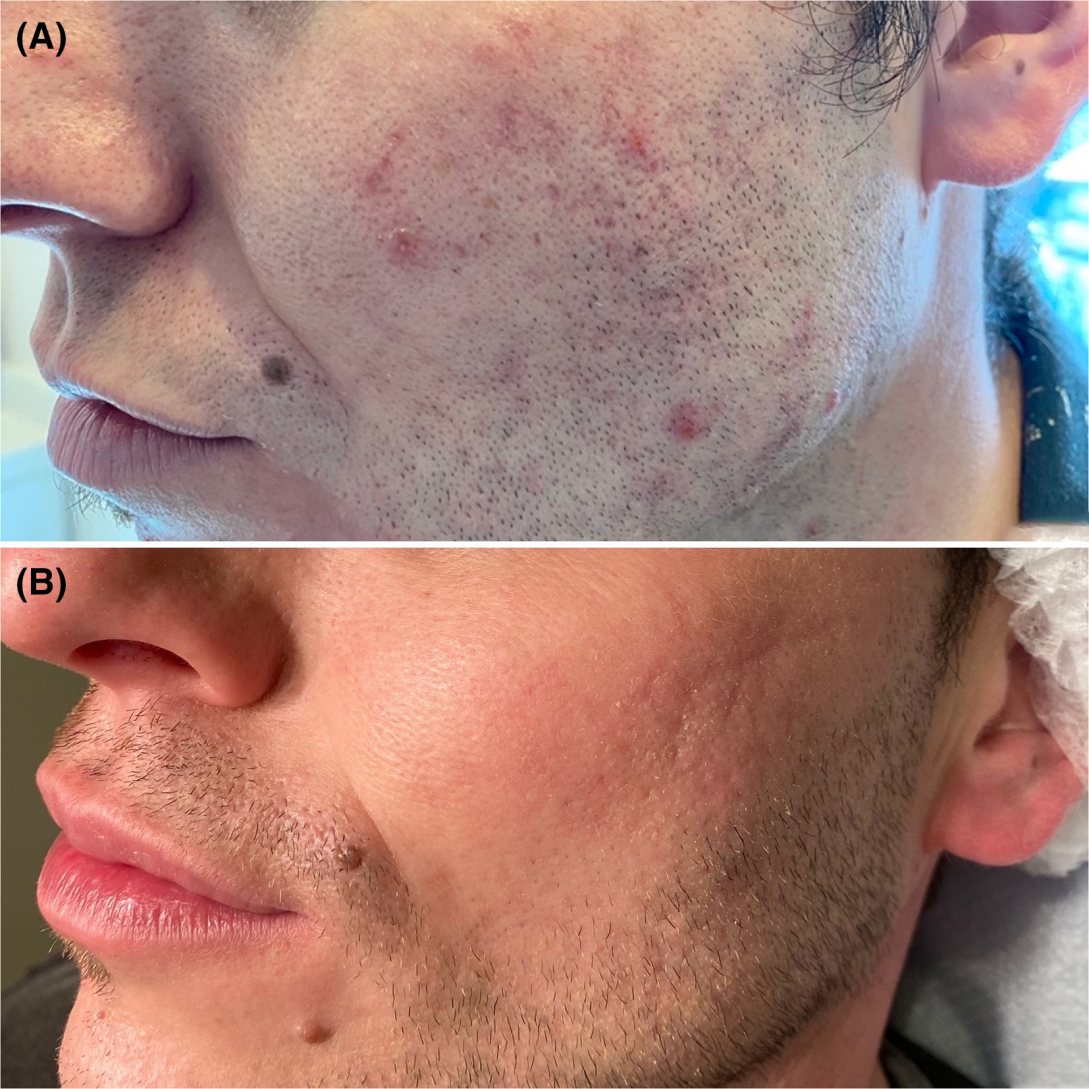
Clinical images before (A) and after (B) treatment with three sessions of IPL and 1550 nm non‐ablative fractional laser. A significant reduction in inflammatory lesions, erythema, and improvement in atrophic scars are observed.

A 33‐year‐old male with severe acne persisting for several years had undergone multiple treatments, including retinoids, antibiotics, and oral isotretinoin therapy. Despite these treatments, inflammatory lesions, erythematous macules, and atrophic scars persisted (Figure 1A).

A new regimen of isotretinoin at 20 mg/day was initiated, and LT was added. Two monthly IPL sessions were conducted using a 530–750 nm filter. In both sessions, an initial application at 7 J/cm^2^ with a double pulse of 2.5 ms and an interval of 10 ms was applied, followed by a second application on specific lesions at 6 J/cm^2^ with 1.5 ms. In the third month, a 1550 nm NFAL session was performed. The initial treatment targeted recessed scars, applying 50 mJ/cm^2^ in 5 ms with 20% coverage using a 4 mm scanner. A comprehensive pass covered the affected region with 35 mJ/cm^2^ in 3.5 ms, providing 25% coverage with a 10 mm scanner. After three sessions of combined LT and oral treatment, there was complete resolution of inflammatory lesions, significant reduction in erythema, and noticeable improvement in scars and skin texture (Figure 1B). The patient reported no complications or adverse effects following laser sessions.

## CASE 2

**FIGURE 2:**
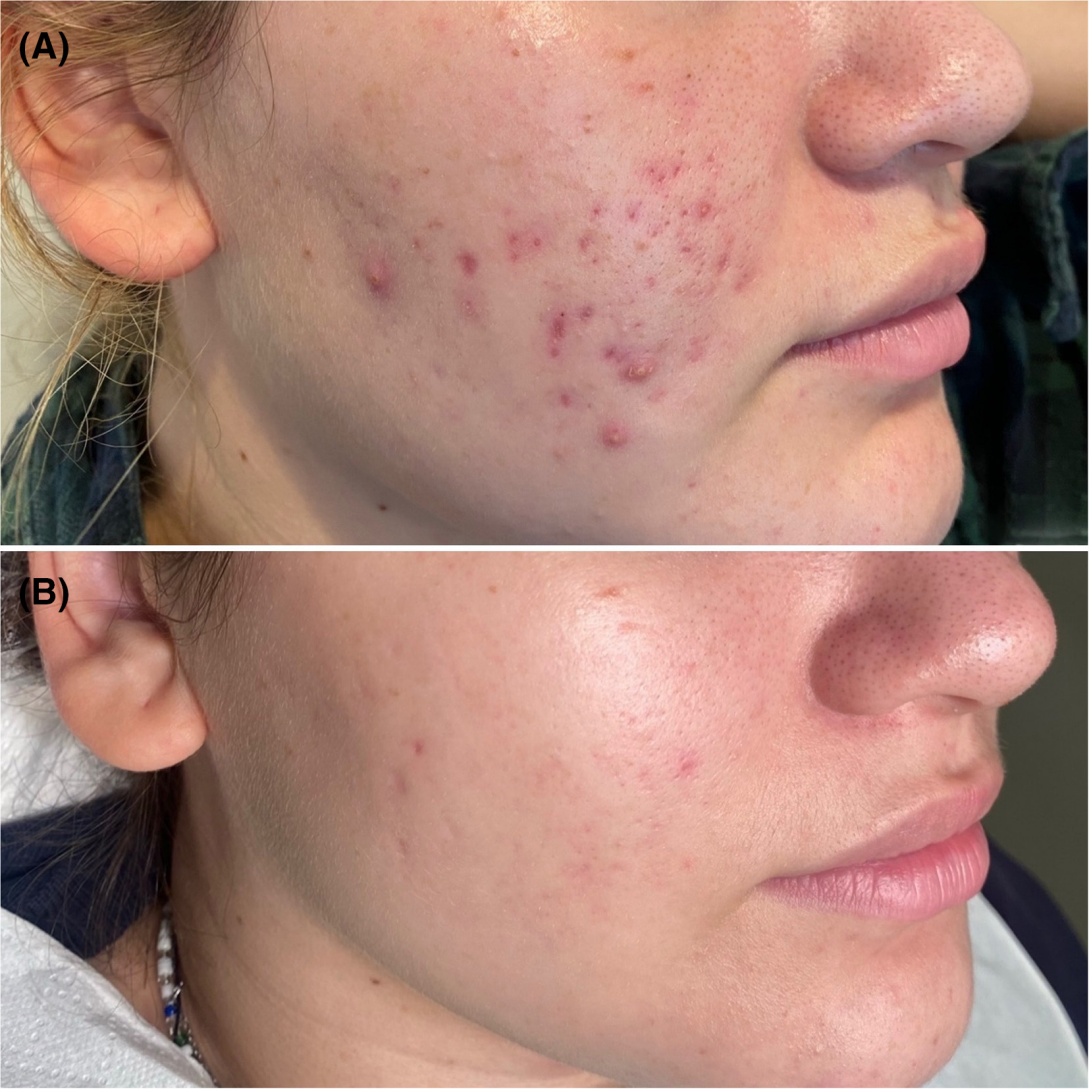
Clinical images before (A) and after (B) three treatment sessions with IPL and 1550 nm non‐ablative fractional laser.

A 16‐year‐old female with severe inflammatory acne showed incomplete response to topical retinoids and a 3‐month regimen of lymecycline 300 mg/day (Figure 2A). The patient and her family declined oral isotretinoin due to concerns about potential adverse effects.

Three treatment sessions combining IPL and 1550 nm NFAL were conducted while maintaining topical treatment. A 530–750 nm IPL filter was employed with a fluence of 7–7.4 J/cm^2^ in a double pulse of 2.5 ms and an interval of 10 ms for a general pass over the affected area. Subsequently, a focal application was performed on specific lesions at 6–6.2 J/cm^2^ in 1.5 ms. Following this, a 1550 nm NFAL was used, with the first focal pass targeting scars at 50 mJ/cm^2^ in 5 ms, covering 25% of the area using a 4 mm scanner. The second pass covered the affected area at 35 mJ/cm^2^ in 3.5 ms with 25% coverage and a 10 mm scanner.

A noticeable decrease in the severity of acne lesions and significant improvement in scar appearance were observed (Figure 2B).

## DISCUSSION

Evidence supports the effectiveness of LT in treating acne and its aftermath. IPL can photocoagulate dermal capillaries and blood vessels, reducing sebaceous gland size, sebum secretion, and post‐acne erythema. It also exhibits bactericidal and anti‐inflammatory effects. Studies support IPL's efficacy in reducing both inflammatory and non‐inflammatory lesions, demonstrating safety and efficacy in monotherapy or combined with systemic therapies like isotretinoin.^2^, ^3^, ^4^

Er:Glass 1550 nm FNAL induces dermal heating and collagen production with less thermal damage in contrast to ablative lasers, generating coagulation columns and respecting the epidermis. This results in shorter recovery periods and reduced postoperative complications compared to ablative lasers.^5^, ^6^

The clinical cases highlight LT's role in managing severe acne, enhancing the response to isotretinoin, reducing treatment duration, and improving patient satisfaction. Combining systemic treatments with LT appears to be an effective strategy for severe acne, particularly for patients unresponsive to conventional therapies. This approach has significant dermatological and psychological benefits, improving self‐esteem and quality of life. However, further research is needed to fully understand the scope and benefits of these therapies, and it is essential for them to be administered by experienced professionals.

## Ethics Statement

The authors declare that they have no competing interests.
